# Efficiency of Diclofenac Removal Using Activated Sludge in a Dynamic System (SBR Reactor) with Variable Parameters of pH, Concentration, and Sludge Oxygenation

**DOI:** 10.3390/ma16041422

**Published:** 2023-02-08

**Authors:** Anna Zając-Woźnialis, Izabela Kruszelnicka, Joanna Zembrzuska, Dobrochna Ginter-Kramarczyk, Marek Ochowiak, Andżelika Krupińska

**Affiliations:** 1 Department of Biophysics, Poznan University of Medical Sciences, Grunwaldzka 6, 60-780 Poznan, Poland; 2 Department of Water Supply and Bioeconomy, Faculty of Environmental Engineering and Energy, Poznan University of Technology, Berdychowo 4, 60-965 Poznan, Poland; 3 Faculty of Chemical Technology, Institute of Chemistry and Technical Electrochemistry, Poznan University of Technology, Berdychowo 4, 60-965 Poznan, Poland; 4 Department of Chemical Engineering and Equipment, Poznan University of Technology, 60-965 Poznan, Poland

**Keywords:** diclofenac, wastewater treatment, activated sludge, SBR reactors, pH

## Abstract

Recently, traditional wastewater treatment systems have not been adapted to remove micropollutants, including pharmaceutical substances, which, even at low concentrations, cause adverse changes in aquatic and terrestrial living organisms. The problem of drug residues in the environment has been noticed; however, no universal legal regulations have been established for concentrations of these compounds in treated wastewater. Hence, the aim of the article was to determine the possibility of increasing the efficiency of diclofenac removal from activated sludge using the designed SBR reactor. This study included six cycles, working continuously, where each of them was characterized by changing conditions of pH, oxygenation, and composition of the synthetic medium. In each cycle, three concentrations of diclofenac were analyzed: 1 mg/L, 5 mg/L, 10 mg/L for the hydraulic retention time (HRT) of 4 d and the sludge retention time (SRT) of 12 d. The highest removal efficiency was achieved in the first test cycle for pH of natural sediment at the level of 6.7–7.0 (>97%), and in the third test cycle at pH stabilized at 6.5 (>87%). The reduced content of easily assimilable carbon from synthetic medium indicated a removal of >50%, which suggests that carbon in the structure of diclofenac restrained microorganisms to the rapid assimilation of this element. Under half-aerobic conditions, the drug removal effect for a concentration of 10 mg/L was slightly above 60%.

## 1. Introduction

Currently, the analysis of environmental samples in terms of the presence of pharmaceutical micropollutants is mainly focused on the most popular groups occurring at the highest concentrations which also include non-steroidal anti-inflammatory drugs [1,2]. Among them, diclofenac (DCF) is a very significant pharmaceutical due to its low degradation capacity [3]. It is an active substance used in a very wide range of medical products available as anti-inflammatory and analgesic pharmaceutical preparations. In 2020, it was the 72nd most prescribed pharmaceutical in the US, with over 9 million prescriptions [4]. Diclofenac is also popular in Poland. According to the data contained in the Register of Medicinal Products, as many as 135 products containing this substance are available on the market [5]. The number of products reflects that diclofenac plays an important role in the pharmaceutical market [6]. Diclofenac is available in both prescription and over-the-counter (OTC) preparations [7]. The universal character of its application and use in medical and veterinary procedures is not indifferent to the natural environment [8,9,10]. It is excreted in the form of metabolites [11,12,13] or transported to the sewage treatment plant as an unused, expired pharmaceutical flushed down the sinks and toilets together with municipal sewage [14]. Currently, there are no legal regulations which require the monitoring or specifying the limit concentrations for diclofenac as a substance toxic to the natural environment, both for drinking water and the composition of treated sewage discharged to water reservoirs. The European Union, at the end of October, published a draft on a proposed Directive amending the Water Framework Directive, the Groundwater Directive and the Environmental Quality Standards Directive [15]. Moreover, the European Union published document No. 2022/0345 (COD) Proposal for a Directive of The European Parliament and of The Council concerning urban wastewater treatment (recast), in which it also includes pharmaceuticals as micropollutants necessary to control [16]. Individual countries, e.g., Hungary, also have their internal effluent quality requirements for pharmaceutical production wastewater [17]. Hospitals and factories producing medicines are also an additional source carrying a load of pharmaceutical impurities [18,19,20]. The Environmental Protection Agency (EPA) promulgated the Pharmaceutical Manufacturing Effluent Guidelines and Standards (40 CFR Part 439) in 1976 and amended the regulation in 1983, 1998, and 2003 [21]. Finally, post-production wastewater is transported to the municipal sewage system or discharged directly to water reservoirs. The level of diclofenac concentration in rivers all over the world ranges from 1 to over 1000 ng/L [14,19]. Toxicity tests indicate that a dose of 5 µg/L exposed for 48 h inhibits the growth of the sea urchin *Paracentrotus lividus* larvae [22].

Due to the recognition of the problem associated with a presence of pharmaceutical compounds in the aquatic environment, the first watch list was implemented in March 2015 based on decision of the Commission of the European Union 2015/4951 [23]. In that document, diclofenac was a representative the group of non-steroidal anti-inflammatory pharmaceuticals as the greatest potential toxic effect [23]. After an analysis of the results of conducted monitoring studies, the European Commission implemented the decision 2018/8402 to remove diclofenac from the watch list [24]. In the communication entitled The Strategic Approach of the European Union to Pharmaceutical Substances in the Environment, issued in 2019, it was estimated that there was a lack of a clear relationship between pharmaceutical substances present in the environment and their direct effects on human health [25,26]. Additionally, based on the materials collected by the World Health Organization, it was confirmed that the probability of being life-threatening for people at low concentrations understood at the level of a therapeutic effect is low [25]. From 21 January 2021, the amended Directive of the European Parliament and of the EU Council No. 2020/2184 on the quality of water intended for human consumption was applied [27]. The directive indicates a new watch list published in the EU Commission Implementing Decision 2022/679 the 19 January 2022, excluding diclofenac [28].

Despite the withdrawal of diclofenac from the watch list, the problem of its presence in the aquatic and terrestrial ecosystem remains. Studies indicate the bioaccumulation of diclofenac in wild aquatic organisms [29], and its metabolites are highly toxic to them [10]; although, the current literature in this field is not yet very extensive. It was initially assessed that the metabolites are more toxic than the parent form of the drug [30]. It is estimated that about 65% of diclofenac is present in the form of metabolites containing the OH group in its structure in various positions, which increases the risk of complexes of these groups with other emerging contaminants (ECs) [11]. Currently, it has been tested that the forms of diclofenac biotransformation in mammals are highly reactive. They induce reactive oxygen species [11], as well as disrupt the endocrine system and have an immunomodulatory effect [31]. In salmonids Salmo trutta treated with diclofenac at concentrations >10µg/L, significant changes in liver and kidney tissues were observed [32]. Balbi et al. (2018) determined the effect of an exposure dose of diclofenac on the marine mussel (mollusc) Mytilus galloprovincialis. An amount of 1 µg/L of diclofenac in water has been shown to affect gene transcription in early developmental stages [33]. Current technological solutions applied in biological sewage treatment plants are not able to effectively remove all residues of this substance. Literature reports indicate that the average efficiency of removing this compound ranges between 20 and 50% [34,35], and in individual cases, it can even reach 90% [36,37]. Residues of diclofenac which are not removed at the stage of biological purification result in its discharge to surface waters, which are often a source of drinking water [38,39]. The disclosure of the problem initiated a number of scientific studies focused on finding technical and material solutions to increase the effectiveness of pharmaceuticals that resistant to degradation [40,41]. The most frequently mentioned alternatives are adsorption [42,43,44], membrane separation [45,46,47], and advanced oxidation processes [48,49,50], as well as hybrid methods [51]. Currently, the highest removal efficiency is achieved by ozonation processes and treatment with activated carbon [52]. However, these methods are susceptible to various limitations. Membrane filtration is highly energy-intensive, adsorption using activated carbon limits its effectiveness in inactivating bacteria, and ozonation can generate harmful by-products [52]. The advanced oxidation process (AOP) is a technology that is able to remove compounds from the group of emerging contaminants (EC) from urban wastewater with high efficiency. However, is not implemented on an industrial scale in relation to the reduction of such pollutants [52]. Alternative methods with high potential for reducing micropollutants include artificial wetlands [53,54], bioelectric systems [55], and enzymatic treatment [56,57,58]. Studies conducted with the use of these technologies have shown great success in removing EC in wastewater treatment plants where they have been implemented [8,52,59].

The current legislative system of the European Union does not specify requirements for the removal of pharmaceuticals from wastewater. In the report of the European Commission for the European Parliament and the Council of 26 February 2019, there is a record indicating the need to carry out activities focused on analyzing the possibilities of reducing pharmaceutical micropollutants. Moreover, it is suggested that there is a need to modernize conventional municipal wastewater treatment plants that will be able to remove these substances, with reasonable economic assumptions [24].

Scientific research confirms that changing the physicochemical parameters, such as pH, temperature, or sludge age, improves the efficiency of pharmaceutical reduction [47,60,61]. The concentration of a given compound and its structural structure is also an important parameter that determines the effectiveness of the degradation of a pharmaceutical compound. An important issue in the design of a technological system focused on removing specific groups or types of pharmaceuticals is also maintaining a high reduction rate of other biogenic compounds contained in wastewater.

The high degree of toxicity of diclofenac and its metabolites among a wide range of NSAIDs [13,62], lack of legal regulations imposing control and reduction of this compound [63], and susceptibility to increased efficiency of diclofenac removal with variable parameters of activated sludge in biological wastewater treatment technologies have become the driving force to conduct the presented research [41].

In order to elucidate the mechanism of diclofenac decomposition and to analyze the use of activated sludge as a material properly stimulated, which can contribute to increasing the efficiency of its removal, an innovative dynamic system in the form of an SBR reactor was prepared. In the six research cycles, the effectiveness of diclofenac removal was analyzed for the determined variable physicochemical values: pH, oxygenation, and variable concentration of the dosed pharmaceutical substance.

The aim of the research was to check the adaptation of the activated sludge to the removal of diclofenac and to assess the effectiveness of its reduction at variable pH values, reduced amount of carbon, as well as reduced oxygen concentration during continuous operation of the reactor. The obtained research results were to point out the possibility of increasing the efficiency of removing this type of compound using conventional biological with activated sludge without the need to develop new technologies and generating additional financial costs for the decay of this substance. Moreover, the ability of the activated sludge to digest the carbon contained in diclofenac as an easily assimilable substrate for the denitrification process was tested. The innovativeness of the research consisted in designing the dynamic SBR system as one long technological process divided into six cycles with different physicochemical conditions. In addition, the size of the research reactor with a volume of 50 L was intended to cope with the maintenance of the process on a large scale, but also the results that can realistically occur on an industrial scale.

## 2. Materials and Methods

### 2.1. Construction of the Test Stand

**Figure 1:**
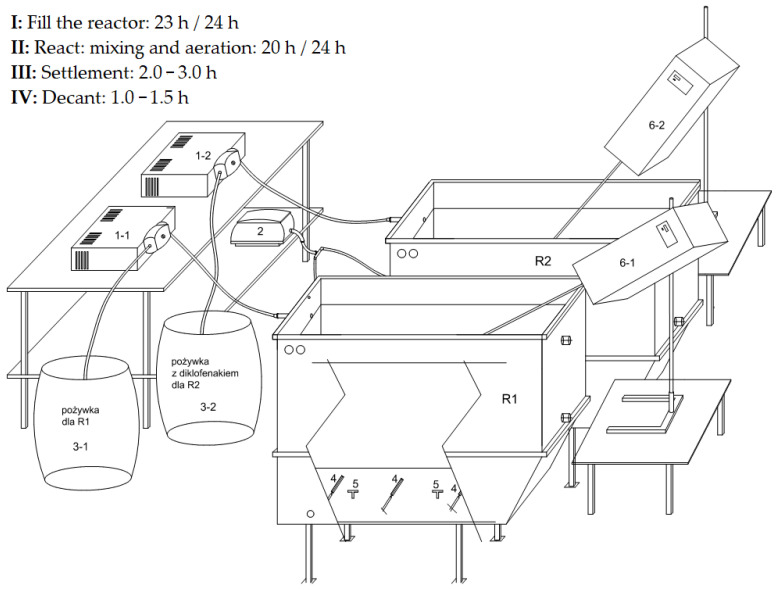
Scheme of the R1 and R2 bioreactor test stand: R1—oxygen bioreactor (reference reactor, without dosing diclofenac); R2—oxygen bioreactor (reactor to which diclofenac was dosed); 1–1—pump dosing the nutrient solution to the R1 reactor; 1–2—pump dosing the nutrient solution to the R2 reactor; 2—blower; 3–1 barrel with diclofenac-free medium dosed to the R1 reactor; 3–2—barrel with diclofenac-containing medium dosed to the R2 reactor; 4—fine-bubble aeration; 5—coarse-bubble aeration; 6–1—R1 reactor stirrer; 6–2-agitator of the R2 reactor.

The effectiveness of diclofenac removal was investigated in a model system of a sewage treatment plant designed and built on a laboratory scale, which is shown in Figure 1. The system consists of two identically operating oxygen reactors with a capacity of 50 L consisting of acid-resistant steel. During the tests, synthetic medium without diclofenac was dosed into the R1 reactor and the individual parameters were changed in accordance with the cycles for the R2 reactor. Therefore, the R1 reactor was a matrix, a reference point for R2 in terms of maintaining the stability of the system operation (e.g., activated sludge growth, color, and odor assessment) to which the drug was dosed. The system has been designed for a long-time work, with constant, defined physicochemical parameters. It is the main element of the research system, which was designed and built on the basis of previously obtained analysis results and a literature review. The starting form of diclofenac, prior to metabolic transformation, was used in the research. The rate of diclofenac removal was assessed using activated sludge grown and adapted to work under specific conditions, collected once from an oxygen chamber at the Central Sewage Treatment Plant in Koziegłowy (near Poznań, Poland). The employed experimental set-up is presented in Figure 1.

### 2.2. Operation of the Test Stand and Equipment

The tests were carried out in parallel using two SBR reactors, designated R1 and R2, made of stainless steel with an active capacity of 50 L. Fine and coarse bubble nozzles were installed at the bottom of the chambers. Air was pumped to the reactors using a blower, model DBMX120 (AirTech, Guangfu Rd, Hukou Township, Hsinchu County, Taiwan), with a capacity of 120 L/min. The R1 reactor was a reference, comparative reactor, in which only synthetic sewage was dosed, without the addition of the active substance—diclofenac. In the R2 reactor, a certain amount of diclofenac was dosed in addition to the synthetic medium. The synthetic medium was transported to the system by means of two dosing pumps, model 153Yx (JIHPUMP, Chongqing, China), with a flow capacity of 0–2.36 L/min at 0.1–600 rpm, separately for each reactor. In addition to R1 and R2 reactors, laboratory mechanical stirrers were introduced—model BMX-15 (Biomix, Mościska, Poland), in order to maintain steady distribution of the medium and added active substance—diclofenac. The reactors were equipped with heaters with a 75 W thermostat, allowing them to maintain a constant temperature at the level of 20 ± 2 °C in the system.

The dynamic SBR system included four main working phases:

In the absence of legal regulations, this solution was focused on verifying the possibility of increasing the reduction of diclofenac and limiting its penetration into the environment. In addition, the research allowed obtaining more information regarding the improvement of the functioning of existing biological wastewater treatment plants without implementing new, costly technologies and to consider it would be possible to implement the proposed solution for the treatment of wastewater generated in plants producing medical products.

### 2.3. Cycles of the Dynamic Test

The research lasted a total of 150 days. It was divided into two stages—introductory and main. The second stage, presented in this article, included the analysis of the diclofenac decomposition dosed into the R2 reactor. The discussed research was conducted from the 62nd to the 150th day and was divided into 6 research cycles (series). The cycles from I to V lasted 12 days, whereas the last, VI cycle of research lasted 4 days. There were 4-day intervals in diclofenac dosing between each series. The first four series involved the investigation of the effectiveness of diclofenac degradation for variable pH values, with the same dosing of 3 concentrations of the drug (i.e., 1.5, 10 mg/L), each for 4 days. The dynamic test was carried out in 6 research cycles, the description of which is presented in Table 1 below. The technological process in the SBR dynamic reactor has been designed for HRT 4d in order to slowly adapt the activated sludge to the diclofenac dosed into the R2 reactor. Sludge retention time (SRT) was set at 12 d due to the fact that wastewater treatment plants (WWTPs) operated at higher SRTs may tolerate and recover from the adverse effects of such micropollutants [64].

### 2.4. The Composition of the Used Medium

The composition of the medium was developed based on Kosjek T. at all 2007 [65]. The final composition is given in Table 2.

### 2.5. LC-MS/MS Analysis

The SPE technique with Oasis HLB cartridges (6 mL, 500 mg, Waters Corporation, 34 Maple Street, Milford, MA 01757, USA) was used for the isolation of diclofenac from the solution after biodegradation experiments. First, an SPE cartridge was conditioned with 5 mL of methanol followed by 5 mL of deionized water, at a flow of 1 mL/min. After the conditioning step, the sample was percolated through the cartridge. After that, the cartridge was dried under a vacuum for 20 min, to remove excess water. For the elution of the sample, 5 mL of methanol was used. The extract was evaporated under a gentle stream of nitrogen and reconstituted in the mobile phase to a final volume of 1 mL for further LC-MS/MS analysis.

The determination of diclofenac was performed using a chromatographic system UltiMate 3000 RSLC (Dionex, Thermo, 1228 Titan Way, Sunnyvale, CA 94088-3603, USA), coupled with an API 4000 QTRAP triple quadrupole mass spectrometer with electrospray ionisation (ESI) (from AB Sciex, Foster City, CA, USA) in negative ionisation mode (UHPLC–ESI MS/MS). A Hypersil Gold C18 RP (100 mm × 2.1 mm, 1.9 μm particle size) column from Life Technologies Polska Sp. z o. o., Warsaw, Poland was used for chromatographic separation of the compound. The temperature in the column of the chromatographic system was maintained at 35 °C and the injection volume was equal to 5.0 μL. For UHPLC–ESI MS/MS analysis, the mobile phase was a gradient prepared from Milli-Q water containing 5 mmol/L ammonium acetate (component A) and MeOH (component B). The following gradient was used: 0 min 30% B, 10 min 67% B, 11 min 100% B, and held for 1 min; the flow rate was 0.2 mL/min. A post-run time was set at 5.0 min for column equilibration before the next injection. The operating conditions for mass spectrometry for diclofenac were as follows: curtain gas, 20 psi; nebuliser gas and auxiliary gas, 40 psi; source temperature, 400 °C; ion spray voltage, −4.500 V; and collision gas set to medium. The quantitative analysis of the compounds was performed in multiple reaction monitoring (MRM) mode. For diclofenac, one transition of deprotonated molecular ion and their respective ion product was chosen. These transitions (*m*/*z*) with associated declustering potentials (V) and collision energies (V) were: 294 → 250, −40, −18; and 294 → 214, −40, −30 [66].

## 3. Results

**Figure 2:**
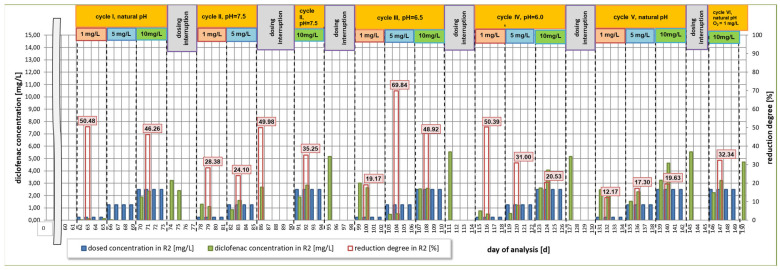
Degree of diclofenac reduction after 1 day from dosing a specific dose of the drug in the R2 bioreactor in all analyzed research series.

**Figure 3:**
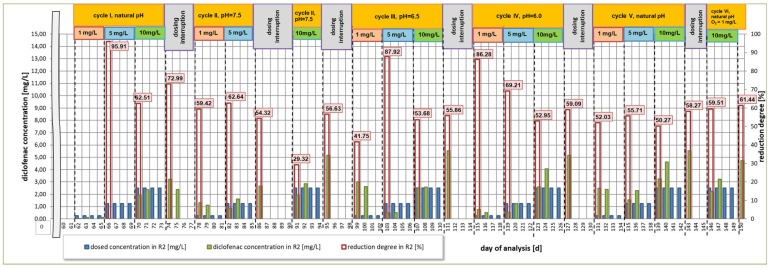
Degree of diclofenac reduction after 4 days from dosing a specific dose of the drug in the R2 bioreactor in all analyzed research series.

**Figure 4:**
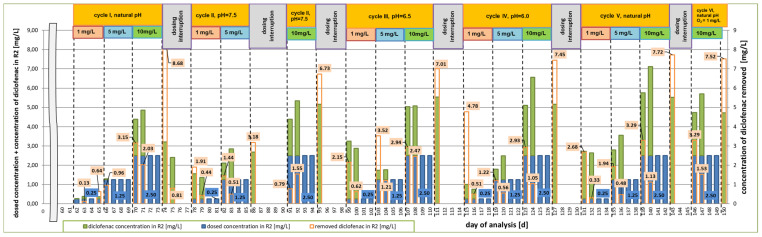
Concentration of the removed diclofenac in relation to dosing the dose of the drug along with its residue in the R2 reactor.

The analysis of changes in terms of diclofenac concentration is shown in Figure 2 and Figure 3, presenting the results of the reduction of its concentration after the first and fourth day following dosing each of the studied concentrations in individual research cycles, respectively. In addition, the efficiency of diclofenac removal in relation to the dosed amount is presented, taking into account the residue of the drug not degraded in previous cycles (Figure 4).

### 3.1. First Research Cycle

The first cycle of research included the analysis of diclofenac degradation applied at three selected concentrations under natural pH conditions. Within the four days during which 1.0 mg/L diclofenac was dosed into the R2 reactor, it was observed that the reduction was equal to 95.9% (Figure 3). The lowest removal efficiency of the analyzed compound (62.5%) was determined after 4 days of dosing at a concentration of 5 mg/L. In case of dosing of 10 mg/L of diclofenac, after the 12th day of the study (74th day of analysis, Figure 3), the obtained rate of reduction was at the level of 73%. On the same day, a 4-day break in dosing to the R2 reactor was started. After this time, before the start of dosing diclofenac for the second series of tests, a 59.4% reduction of the drug was observed compared to the value on the last day of the series I.

### 3.2. Second Research Cycle

During the second cycle, starting on day 78 of the test, the pH was maintained at 7.5. At the end of the dosing of diclofenac at a concentration of 1.0 mg/L, the reduction was equal to 62.6%. After another 4 days of dosing at 5 mg/L, the removal was equal to 54.3%. This may be caused by the fact that on the day of the start of the second series of tests, the concentration of diclofenac in the R2 reactor was at 1.3 mg/L, the presence of which could limit the decomposition of the next dose of the compound. After the 5 mg/L dosing was completed, there was a five-day break in drug dosing into the system. On the first day of dosing 10 mg/L of diclofenac into the system, it was observed that the drug concentration in the R2 reactor reached the same value as in the first cycle (1.89 mg/L). After the dosing of the active substance at the highest concentration was completed, the reduction rate was at the level of 56.6%. Despite a slight change in pH, compared to the first cycle, the reduction of diclofenac decreased by more than 16%. This may suggest that even a slight increase in pH (less than 0.5) inhibits the degradation of diclofenac, which was confirmed by Kimura et al. [67]. The sludge index during the first eight days of the second cycle maintained similar values as in the first cycle. During the dosing period of the highest concentration, a decrease in the sludge index value from over 320 mL/g (85 day of analysis) to 237 mL/g (95 day of analysis) was observed in relation to the R1 reactor. This may suggest that the presence of diclofenac does not have a toxic effect on activated sludge microorganisms. After completion of the second cycle, there was a break in the dosing of diclofenac from days 95 to 98. Moreover, the R2 reactor was prepared for the next series of tests, stabilizing the pH in the system at a level of 6.5. A residual drug reduction of 41.7% compared to the value from the last day of the second series of tests was observed.

### 3.3. Third Research Cycle

In the third cycle, which lasted from the 99th day of the test (Figure 2), the total reduction of the drug after dosing the concentration of 1 mg/L was equal to 87.9%, which is higher by 25% than the value obtained at pH = 7.5 despite the very high diclofenac residue at the beginning of the cycle (approx. 3 mg/L). The reduction of the active substance was noted at a similar level in both cycles after introducing doses of 5 mg/L and 10 mg/L into the system, taking into account similar values of residues. A higher value of the index was recorded on days 101 to 103 (period of dosing diclofenac at a concentration of 1 mg/L) and on day 108, after the first day of dosing diclofenac at a dose of 10 mg/L. The lowest diclofenac reduction rate of 53.7% was also obtained at this time. After the completion of the third research cycle, in which the experiment was focused on analyzing the course of diclofenac reduction for the sewage pH of 6.5, a 4-day break in drug dosing was started. During its duration, the pH in the reactors was lowered to 6.0. The value of this indicator was maintained throughout the fourth research cycle.

### 3.4. Fourth Research Cycle

Based on the obtained results, a decrease in pH by 0.5 leads to a lower reduction of diclofenac at a dose of 1 mg/L (69.21%). The removal efficiency is more than 18% lower compared to the pH 6.5 experiment, even though the drug residue was similar. The degradation efficiency of the active substance for doses of 5 and 10 mg/L was 53% and 59%, respectively. These values were very similar to the previous cycle at the same time and amounted to 53.7% and 56%, respectively (Figure 3). The microscopic analysis carried out on the last day of this series showed good sedimentation properties of the activated sludge. Medium-sized, light brown flocs were predominant. In the system, however, there is no great diversity within microorganisms. After the end of the fourth cycle, a four-day break in diclofenac dosing was performed. During this period, the pH developed naturally in relation to the changes occurring in the system, without pH correction.

### 3.5. Fifth Research Cycle

In the fifth cycle, the amount of organic carbon in the synthetic medium was reduced by 5% compared to the amount of carbon in the starting medium. This assumption was focused on examining whether the reduced value of this element, which is the main component of the medium, will be compensated by the organic carbon contained in the structure of diclofenac, and, thus, if it will be possible to achieve a greater effectiveness of its degradation. As a result, the average removal efficiency was equal to 50% for all applied doses. With a small percentage difference, the highest diclofenac removal efficiency was obtained when dosing the highest dose of 10 mg/L. The microscopic analysis carried out two days before the end of the tests showed an improvement in the functioning of the activated sludge in relation to the cycle conduced at pH = 6.0. During a 4-day break, after the completion of the fifth research cycle, the oxygen concentration was lowered to a value of 1.0 mg/L. The last, sixth research series was carried out additionally, only for the concentration equal to 10 mg/L and at a reduced level of oxygen concentration of 1.0 mgO_2_/L. In this test cycle, the degradation of diclofenac was observed at a level of 61.44%.

### 3.6. Statistical Analysis

Statistical analysis of test results was carried out for three configurations:

1. Comparison of the test results of selected parameters, determined throughout the period of diclofenac dosing (i.e., from 62 to 150 days) in the R2 reactor, with the results determined for these parameters in the R1 reactor;
2. Comparison of the test results of selected parameters for each research cycle, including the dosing of three concentrations of diclofenac to the R2 reactor (i.e., cycles I–V) in relation to individual research series carried out in parallel in the R1 reactor;
3. Comparison of the test results for each of the selected parameters analyzed in the first cycle (adopted as a model) in relation to the remaining second to fifth research cycles, in the R2 reactor, during the dosing of diclofenac.

**Figure 5:**
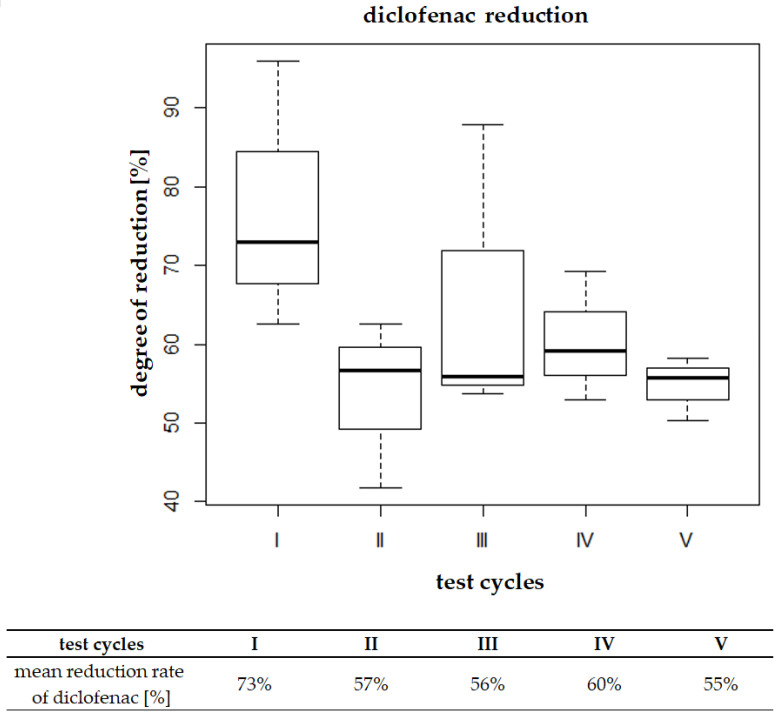
Distributions of diclofenac reduction concentration in cycle I of diclofenac dosing in reactor R2 in relation to individual research cycles.

The results of statistical analyses were carried out in the R program (R Core Ream, 2017) [68]. The practical interpretation of the test results was carried out on the basis of the obtained *p*-values from the individual tests. In order to graphically represent the test results, box-whisker plots were made. The obtained results of diclofenac reduction are presented in Figure 5.

Analysis of the data presented in Figure 5 indicates that the average degradation of diclofenac in cycles II–V was equal to 55–60%, regardless of the dosed dose. The highest reduction percentage was obtained in the first test cycle, at natural pH in the bioreactor. The high reduction in the first series of tests may result from favorable conditions for the activated sludge and stable operating parameters in the system at the stage of incorporation of the activated sludge. In each of the subsequent cycles, during dosing of a fixed concentration of diclofenac, residues of the analyzed substance from the series preceding the next cycle were present in the R2 bioreactor. Thus, the presence of diclofenac in the system during the application of the next dose could further deteriorate the efficiency of its removal.

## 4. Discussion

The objective of this research was to investigate the efficiency of drug reduction by activated sludge microorganisms as a function of changing pH value in the range from 6.0 to 7.5, at short intervals. Irrespective of the adopted variant, it can be observed that the highest rate of diclofenac reduction occurred at the pH value within the range of 6.5–6.7–7.0, observed in the first and third research cycles. In both cases, the results were obtained after the fourth day of dosing diclofenac at a concentration of 1 mg/L (Figure 3). Even within the same activated sludge biological removal method, different mechanisms for the degradation of pharmaceutical contaminants are distinguished. The literature indicates that some compounds, including diclofenac, are removed by biotransformation, and, to a minor extent, by adsorption, hydrolysis, or oxidation [30,69]. Other studies indicate the dominant role of sorption processes [8,70]. With regard to the dynamic system, in order to check whether the activated sludge microorganisms can use diclofenac as a source of organic carbon, the amount of carbon in the medium used during the fifth cycle was reduced by 5% compared to the initial composition. However, the obtained results indicated the lowest efficiency of drug removal compared to all other research cycles (Figure 2, Figure 3 and Figure 4). This suggests that the microorganisms, as a result of the deficit of an easily available source of organic carbon contained in the starting medium, tried to adapt to the new conditions, but were unable to compensate for the loss of this element with carbon available in the structure of diclofenac. One of the reasons for the difficulty in removing diclofenac may be the presence of a chlorine atom in its structure, which preserves the drug presence in treated wastewater [30].

The literature indicates that the efficiency of diclofenac removal by conventional biological wastewater treatment plants with chemical phosphorus removal reaches 20–30% [71,72]. Treatment plants with SRT around 10 d achieved a removal of 70–75% [73,74]. The diclofenac removal results obtained in the studies in cycles I and III are higher (87–97% for pH 6.5–7.0). Other values range from 22 to 75, at a level similar to that in other test cycles (however, for higher values of dosed concentrations).

Diclofenac is acidic, which is confirmed by the value of the pKa = 4 [75]. In research of Yan when pH 4.15–7, diclofenac coexisted in the anionic form and the molecular form. Then, the anionic form of DCF repelled the sludge, and the adsorption amount slowly decreased. The adsorption of DCF is affected by its hydrophobicity (neutral form), and the amount of adsorption decreases with the increase of pH value [70]. This means that beyond the pH value of 4, the ionized form of diclofenac will dominate in the solution. This form is more friendly to bacteria. Hence, running the wastewater treatment processes in the presence of diclofenac above the pH value of 4 seems to be more beneficial. The decomposition of diclofenac above pH = 4 was also noticed in the adsorption process on activated sludge [8]. This assumption was confirmed by the results of the diclofenac reduction rate, which was equal to 95% after only 4 days of the degradation process during the dynamic test at an average pH of 6.7–7.0 for the initial diclofenac concentration of 1.0 mg/L. More than 87% reduction was also observed in the third cycle, in which the pH value was set to 6.5. The recorded value also occurred after the end of dosing of diclofenac at a concentration of 1 mg/L. According to the obtained test results, discontinuation of cyclic dosing of the analyzed drug for the pH value within the range of 6.0 may increase the efficiency of the removal of this compound. The reduction of diclofenac during the break after the end of the third cycle of research supports this statement. The content of diclofenac on the fourth day of the pause period decreased by more than 86% compared to the first day. Diclofenac was not completely removed, but lowering the pH significantly increased its degradation efficiency. The conducted research allows us to conclude that modification of the physicochemical parameters prevailing in conventional wastewater treatment plants allows increasing the effectiveness of diclofenac removal. Despite the reduced efficiency of diclofenac removal in the anaerobic reactor, the result for the highest concentration of 10 mg/L was 61.5% for natural pH (compared to 73% aerobic efficiency for the same pH). Scientists revealed that the right selection of technology, parameters, and appropriate microorganisms can also be an effective way to remove pharmaceutical contaminants [76]. Regular dosing of diclofenac to the anaerobic treatment process carries the risk of reducing gas production and the dentification potential [60]. Due to the large variety of pharmaceutical substances, as well as their different biotransformations, depending on the environment in which they are found, it seems that achieving a total reduction of pollution is possible only by combining various technologies, the so-called hybrid solutions [77,78].

In the economic aspect of the implementation of the proposed solutions, the research concept focused on the lack of generating additional costs in the removal of diclofenac. One of the basic assumptions was a wide knowledge and use of the degradation possibilities of activated sludge in order to avoid the need to apply additional costs for the construction or extension of the existing conventional methods of wastewater treatment, whereas, in the economic aspect of conducting the research experiment, due to the size of the reactor scale and the duration of the analyses, it required large financial outlays related to the current purchase of reagents for analysis and culture medium and laboratory glass, as well as infrastructural fees related to the operation of the system.

Strengths of the research: the undertaken experiment allowed us to conduct research in two parallel SBR reactors with a large volume of 50 L, lasting continuously for 3 months, 7 days a week. The purpose of the increased volume of synthetic effluent with cultured activated sludge was to obtain feasible results in relation to the results expected on a real industrial scale. The research was focused on variable pH values and oxygenation, in view of the fact that these are some of the most frequently controlled parameters in terms of the correctness and effectiveness of biogenic compounds removal using activated sludge in a wastewater treatment plant. The assumed values of diclofenac concentrations dosed into the system are much higher than those currently analyzed in the aquatic environment. The values used made it possible to verify the effectiveness of activated sludge in reducing this drug for increasing concentrations in the environment, caused, for example, by its accumulation or as a residue after increased production of the drug from pharmaceutical plants. The system enables the installation of new devices and elements in order to refine research in the field of removing pharmaceutical compounds using biological methods.

Weaknesses of the research: the SBR system was not controlled automatically and remotely. Aeration parameters, pH, and temperature were manually controlled (correct operation of the system was made three times a day at intervals of about 3–4 h). Due to the size of the reactor scale and the duration of the analyses, the conducted experiment required a larger number of reagents, as well as constant access to a water source or power supply.

## 5. Conclusions

The studies carried out indicate the possibility of increasing the reduction of recalcitrant diclofenac by means of conventional biological methods of wastewater treatment, assuming the modification of basic physicochemical parameters such as pH or degree of oxygenation. Significant parameters which determine the rate of its reduction are the concentration of the toxic substance, as well as its structure and properties. Taking into account the modifications of individual parameters to increase the efficiency of removing a given toxin, an important element is to maintain the correct ratio of biogenic compounds and other parameters responsible for the efficiency of the entire wastewater treatment system.

## Figures and Tables

**Table 1:** Description of individual research cycles of the SBR reactor operation.

Cycle Number	Description of the Performed Activities	Established Working Parameters of the System	
0	Development and adaptation of activated sludge to laboratory conditions, as well as ensuring constant operating conditions of the system at a specific temperature.	T (°C) pH (-) O_2_ (mg/L) SRT ^1^ (d)HRT ^2^ (d)	20 ± 2 °C pH: 6.5–7.5 2 ± 1 mg O_2_/L 12 d 4 d
I	Analysis of diclofenac removal efficiency for a specific concentration (C_DCL_—mg/L). Maintenance of specified operating conditions in both reactors. Dosing of the medium to reactors.	T (°C) pH (-) O_2_ (mg/L) SRT	20 ± 2 °C pH: 7.0–7.5 (natural) 2 ± 1 mg O_2_/L 12 d
		HRT (d)/(C_DCL_—mg/L) time period (d)	4 d for 1 mg/L/5 mg/L/10 mg/L 12 d
II	Analysis of diclofenac removal efficiency for a specific concentration (C_DCL_—mg/L). Maintenance of specified operating conditions in both reactors. Dosing of the medium to reactors.	T (°C) pH (-) O_2_ (mg/L) SRT	20 ± 2 °C pH: 7.5 2 ± 1 mg O_2_/L 12 d
		HRT (d)/(C_DCL_—mg/L) time period (d)	4 d for 1 mg/L/5 mg/L/10 mg/L12 d
III	Analysis of diclofenac removal efficiency for a specific concentration (C_DCL_—mg/L). Maintenance of specified operating conditions in both reactors. Dosing of the medium to reactors.	T (°C) pH (-) O_2_ (mg/L) SRT	20 ± 2 °C pH: 6.5 (natural) 2 ± 1 mg O_2_/L 12 d
		HRT (d)/(C_DCL_—mg/L) time period (d)	4 d for 1 mg/L/5 mg/L/10 mg/L12 d
IV	Analysis of diclofenac removal efficiency for a specific concentration (C_DCL_—mg/L). Maintenance of specified operating conditions in both reactors. Dosing of the medium to reactors.	T (°C) pH (-) O_2_ (mg/L) SRT	20 ± 2 °C pH: 6.0 (natural) 2 ± 1 mg O_2_/L 12 d
		HRT (d)/(C_DCL_—mg/L) time period (d)	4 d for 1 mg/L/5 mg/L/10 mg/L12 d
V	Analysis of diclofenac removal efficiency for a specific concentration (C_DCL_—mg/L). This cycle assumed the analysis of the effectiveness of diclofenac removal, with the amount of carbon in the starting medium reduced by 5%.	T (°C) pH (-) O_2_ (mg/L) SRT	20 ± 2 °C pH: natural 2 ± 1 mg O_2_/L 12 d
		HRT (d)/(C_DCL_—mg/L) time period (d)	4 d for 1 mg/L/5 mg/L/10 mg/L12 d
VI	Analysis of diclofenac removal efficiency for a specific concentration (C_DCL_—mg/L).Reduction of oxygen concentration in reactors. Dosing of the medium to reactors.	T (°C) pH (-) O_2_ (mg/L) time period (d)	20 ± 2 °C pH: natural 0.5–1 mg O_2_/L 4 d
		HRT (d)/(C_DCL_—mg/L)	4 d/10 mg/L

^1^ sludge retention time (SRT), ^2^ hydraulic retention time (HRT).

**Table 2:** The composition of the medium selected and used in the research.

Component	Concentration [mg/L Medium]
Casein peptone (CAS:91079-40-2) Sigma-Aldrich/Merck (Darmstadt, Germany)	160
Meat extract (70164), Fluka Chemie (Buchs, Switzerland)	110
Urea CO(NH_2_)_2_ (CAS 57-13-6) Chempur (Piekary Śląskie, Polska)	30
K_2_HPO_4_ (CAS 7758-11-4) Chempur (Piekary Śląskie, Polska)	28
NaCl (CAS-7647-14-5) Chempur (Piekary Śląskie, Polska)	11
Glucose—C_6_H_12_0_6_ (CAS 50-99,7) Chempur (Piekary Śląskie, Polska)	114
Diclofenac sodium salt C_14_H_10_Cl_2_NNaO_2_, (CAS:15307-79-6) Sigma-Aldrich/Merck (Darmstadt, Germany)	Dosed into the R2 reactor in the amount of 1, 5, or 10 mg/L
distilled water	refill to 1 L

## Data Availability

Not applicable.
